# Association Between Self-Rated Political Orientation and Attitude Toward the Cash Transfer Policy During the COVID-19 Pandemic: A Nationwide Cross-Sectional Survey Conducted in South Korea

**DOI:** 10.3389/fpubh.2022.887201

**Published:** 2022-05-17

**Authors:** Jin-Hwan Kim, Deok Hyun Jang, Won Mo Jang

**Affiliations:** ^1^Department of Public Health, Graduate School of Public Health, Seoul National University, Seoul, South Korea; ^2^Research Analytics and Communications, Gallup Korea, Seoul, South Korea; ^3^Department of Public Health and Community Medicine, Seoul Metropolitan Government – Seoul National University Boramae Medical Center, Seoul, South Korea

**Keywords:** political orientation, social policy, COVID-19, cash transfer policy, health policy

## Abstract

**Objective:**

This study assessed the relationship between self-rated political orientation and attitude toward the cash transfer policy during the COVID-19 pandemic.

**Methods:**

This cross-sectional study conducted in South Korea during the pandemic included a stratified sample of 1,004 respondents (aged 19 years and older). We tested the hypotheses that political orientation shapes attitudes toward social policies and that other socioeconomic factors might have relatively minor importance. Logistic regression was used to identify associations between political orientation and attitude toward the cash transfer policy. Average marginal effects were calculated to determine the effect size of each variable.

**Results:**

Political orientation, age, and residential area were correlated with attitudes toward the policy. Compared to the conservatives, the non-committed and the moderate showed about 10% more favorable attitudes, and the progressive group showed robust support. People in their 30s and 40s showed similar attitudes to the 18–29 group, while older people showed much lower support. Compared to the Seoul metropolitan area, residents of the Ho-nam area showed favorable attitudes, and those of the Yeong-nam area had relatively unfavorable attitudes.

**Conclusions:**

This study suggests that attitudes toward the cash transfer policy are mainly associated with political orientation. Although these results illuminate pandemics' social and political dimensions, further efforts are needed to fully understand the determinants and mechanisms of attitudes toward policies outside the traditional health policy scope.

## Introduction

The Coronavirus Disease 2019 (COVID-19) pandemic has created a severe global crisis. Through the many epidemics that have occurred throughout recorded history, we have learned that the best method of combating novel infectious diseases for which there are no drug- or vaccine-based treatments is restricting societal interactions, a practice commonly known as “social distancing,” “physical distancing,” or “non-pharmaceutical intervention” (1, 2). During the COVID-19 pandemic, countries worldwide recognized the effectiveness of social distancing for preventing the spread of the virus (3); however, such societal restrictions can have negative economic impacts. South Korea has recently experienced the economic effects of addressing a severe epidemic; in 2015, the Korean government created a revised supplementary budget of 11.8 trillion Korean won to boost the economy after being depressed by a 69-day Middle East respiratory syndrome (4). However, because of its much larger scale and impact, the situation relating to COVID-19 is quite different. Countries worldwide are injecting vast amounts of liquidity into their economies, representing dozens of percent of gross domestic product. Several studies published in leading health and medical journals have highlighted the pandemic's effects on countries' economies as an important issue (5, 6). The large-scale effects of the current pandemic have created an unprecedented situation. The social distancing and lockdown policies implemented to combat COVID-19 have negatively affected many people's economic lives (7, 8), and government's fiscal stimulus packages have consequently included direct cash transfers to stimulate consumption and supplement people's living expenses (9).

The Korean government was initially reluctant to increase fiscal spending during the COVID-19 pandemic (10) but, after lengthy discussion regarding the cash transfer scope, amount, and nature, finally decided to provide direct cash transfer unconditionally at the end of April 2020 (11, 12). The fund was distributed at the household level on a one-off basis. The amount of money offered depending on the number of members in each household (households with more than three people received approximately $890 US dollars, three-person families received around $710 US dollars, two-person families received about $530 US dollars, and single-person families received about $355 US dollars) (13); the fund was provided in the form of hard cash (for emergency aid only), credit/check cards, local gift certificates, or prepaid cards (14). The government tried to maintain residualism by encouraging high-income earners to donate funds voluntarily. Eventually, a *de facto* unconditional cash transfer was made, as just two percent was donated (15).

South Korea's first cash transfer, made in May 2020, had some distinctive features. First, it was universal, which differentiated it from subsequent cash transfers (means-test-based), and this rekindled a long-standing debate between universalism and residualism (16, 17). Many people, including leading politicians, extensively argued that the unconditional cash transfer was a form of basic income (12, 18). The politically progressive nature of the policy had been further strengthened in the process. During the debate, the government continued its retreat from its previous position (i.e., from having no plan to provide cash transfers to agreeing to make conditional provisions) by changing its approach from providing selectively (19) to providing universally (14). Second, the COVID-19 pandemic has a societal quality. It impacted the general public of South Korea; as an infectious disease epidemic, it is classified in South Korea as a social disaster, and various studies have examined its social dimensions (20, 21). Consequently, the cash transfer policy represents the government's policy for responding to the societal dimension of the pandemic, and the associated decision-making process was also societal in that it was influenced by pressure placed on the hesitant government by public opinion (22). Third, the social conversation and decision-making regarding the cash transfer policy occurred timely close to the general elections held on April 15, 2020. As a result, the first cash transfer policy had a more political nature than the subsequent cash transfers.

In general, public attitudes toward fiscal policy are affected by individual characteristics such as age, education, gender, race (23–25), personal economic well-being, confidence in politicians, and political ideology (26, 27). Similar to the general attitude toward fiscal policy, it is also well-known that policies to cope with the COVID-19 pandemic are also affected by gender, message (28), partisanship (29, 30), and national identity (31). The relationship between cash transfer policy and political ideology is somewhat vague compared to general fiscal policy. A good starting point for a cash transfer policy is Bolsa Familia, the world's most extensive cash transfer policy. Bolsa Familia is a conditional cash transfer program, but it is closely related to the rule of the left-wing government because of its financial size. Considering that Mexico, the first country to introduce a conditional cash transfer program with Brazil, had taken a different path from Brazil, the issues should be divided into the adoption of policy and the strictness of conditionality (32). The experiences from Latin America indicate that the adoption of conditional cash transfer policy is not by political orientation but by the technocrats who internalized the common understanding of the international development communities and politicians who sought domestic political interests (33). Unlike the adoption of cash transfer policy as knowledge power combines with the interest of political power, its generosity seems to be linked to political ideology (32). At this point, it can be understood that the favor for unconditionality is connected to the pursuit of basic income. This closeness between unconditional cash transfer and basic income suggests that the left (34) and the younger generation (35) generally support the unconditional cash transfer policy.

As Blinder and Krueger's seminal work shows, the influence of ideology is overwhelming compared to other individual factors (27). People go against its power only if the infringed profits are large enough; otherwise, they generally hold an ideologically-based attitude (36). In addition to the theoretical background on public attitudes toward general fiscal policy, the following contextual factors make us predict that political orientation would be the most important: 1) the cash transfer policy would not impose the personal penalty if some are in favor or against the policy contrary to income tax elevation; 2) benefits from the policy would not be large enough, as the pandemic did not last long enough for the general public to feel substantial suffering from income loss; 3) the policy's progressive nature was strengthened as the relationship with universal basic income (UBI) was raised in the policy-formulation process. Age and region also affect attitudes toward the policy, as they are political variables separate from self-reported political orientation. Generally, people in their 50s, the mainstream of society, and those in their 60s, the industrialization generation, are conservative, while those in their 30s and 40s are relatively progressive in South Korea (37). The UBI-like nature of the policy also indicated that younger people would be more favorable (35). The identities of regions have been a much more critical political axis than those of generations in South Korea. There have been ups and downs due to changes in the political situation, but Ho-nam is still the political foundation of the Democratic Party of Korea, a centrist liberal party, and Yeong-nam of the People Power Party, a right-wing party; other regions tend to vote differently depending on the political environment (38).

Considering that the pandemic invoked fear of the unknown, risk perception might influence the policy. Risk perception would have bidirectional effects as a mediating variable between political orientation and perspective toward the policy. People who perceive risk easily usually prefer safer options (36, 37) and are likely to be politically conservative (39, 40). However, there is evidence that one's political view modulates risk perception, especially in emerging populism (41); the timing of this survey suggested a low level of overall perceived risk, making this the more likely option.

Differences can occur for people with a similar political orientation depending on their level of income loss and their COVID-19-related risk perception, even though political orientation mainly determines the attitude toward the cash transfer. We evaluated the interactions between political orientation and income loss, and risk perception to reflect this possibility. Similarly, the interaction between income reduction and risk perception was further assessed to identify the overlapping effects of COVID-19 within a person.

Based on the existing knowledge and policy context, we explored the relationship between self-rated political orientation and attitude toward the cash transfer policy. We investigated the correlation of other potentially associating factors (e.g., perceived risk of COVID-19 infection, income changes during the pandemic) using survey data collected immediately before the provision.

## Materials and Methods

### Participants

A total of 1,004 participants aged 19 years and older were surveyed on May 6 and 7, 2020. The survey was conducted using random digit dialing. Samples were selected after stratification by age, sex, and province. Of the 19,551 eligible cases, 7,147 were contacted (contact rate 3: 36.6%), of whom 1,004 responded (cooperation rate 2: 14.0%, response rate 5: 5.1%) (see Supplementary Materials S1, S2 for survey details and interview guide) (42, 43). Weighting was used to ensure that the sample represented the general population. The weights were used only to calculate proportions and ratios, not to estimate the number of subpopulations. Trained interviewers conducted all interviews through computer-assisted telephone interviewing. The survey was conducted by Gallup Korea, an affiliate of Gallup International.

The demographic factors assessed in the survey included gender, age, occupation, self-reported household economic status, residential area, income change during the COVID-19 pandemic, and political orientation. Age was classified into five levels (18–29, 30–49, 50–59, and 60 years and older). Occupation was organized into seven types: “unemployed,” “farming/forestry/fishery,” “self-employed,” blue-collar worker,” “white-collar worker,” “full-time homemaker,” and “student.” Self-reported standard of living was classified as “lower,” “lower-middle,” “middle,” “upper-middle,” and “upper.” The standard of living was measured as subjectively recognized status without reference point. We later reclassified “lower” and “lower-middle” as “lower,” “middle” as “middle,” and “upper-middle” and “upper” as “upper.” Residential areas were recorded in terms of the province and, referring to political-science literature on local politics in South Korea, were grouped into five large regions, which included provinces and other areas: Seoul metropolitan area, Chung-chung, Ho-nam, Yeong-nam, and Gangwon/Jeju (38). Income change during the COVID-19 pandemic was initially assessed as “decreased,” “no change,” and “increased”; however, since only 12 respondents reported “increased,” the categories were reclassified as “decreased” and “no change or increased.”

### Survey Instruments

Political orientation was recorded using five levels (“very conservative,” “conservative,” “moderate,” “progressive,” and “very progressive,” respectively) according to the subjective evaluation of the respondents, and was then reclassified into “conservative,” “moderate,” and “progressive.” Each person uses somewhat different standards to describe themself as progressive or conservative; nevertheless, in South Korea, progressive ideology is associated with economic individualism, political equality, and a less hawkish attitude toward North Korea (44). The number of people who answered “don't know/refuse to respond” for the political-orientation item was relatively large (166 out of 1,004 respondents), and this answer provided some indication of these people's political stance (45–47). This group, which usually accounts for about 15% of respondents in other polls by Gallup Korea, is more of a cover-up of their political orientation than a lack of political orientation. There might be various reasons for causing a kind of shame in breaking away from one's political orientation group. Considering the survey timing when the approval rating for the ruling party was very high due to the successful COVID-19 response, this group might be conservative but have some difficulty expressing their political stance. The combined proportion of conservative respondents and this group was 37.3%, similar to the proportional representation vote (40.6%) obtained by the two conservative parties in the previous general election. They generally move toward the center of the political horizon and virtually act like the moderate group regardless of their original position. For this reason, unlike other survey items, we included the “don't know/refuse to respond” responses for the political-orientation item in our analysis. Attitude toward the cash transfer policy was evaluated using the question, “The government has decided to provide funds ranging from 4,00,000 KRW for single-person households to one million KRW for households with four or more people. Do you think that this is a good policy or a bad policy?” Responses to this question were recorded as “good” or “bad.” Considering the nature of the survey, this item asks whether the national cash transfer policy is approved of in its entirety. Therefore, the support implied in the answer “good policy” ranges from solid support (i.e., right in every aspect) to weak support (i.e., there are many problems, but it is a good thing overall).

Risk perception regarding a contagious disease may critically influence people's motivation to cooperate in the overall pandemic response (48, 49). For this reason, we also investigated risk perception's effect on perceptions of the policy, which is considered a relatively remote topic compared to social distancing and compliance with personal hygiene rules. Affective and cognitive risk perceptions were evaluated separately. We assumed that risk perception would not affect attitudes toward the policy; however, affective risk perception might have a more favorable impact than cognitive risk perception on the attitude toward the cash transfer policy if the effect exists because the former responds faster to changes in circumstances and lasts longer than the latter (see Supplementary Material S3 for details regarding the risk-perception items applied) (50). The responses concerning income change during the COVID-19 pandemic and political orientation were suggested several times, changing the ordering of the potential reactions.

### Analysis

Response rates for each item were calculated in terms of attitudes toward the cash transfer policy, and univariate analyses using chi-square tests were performed to measure differences between positions on the cash transfer policy for each response. The proportion of missing values for each variable is presented in Supplementary Table S1. These were omitted only when statistics were calculated without being removed; we did not go through the process of identifying and removing outliers. Attitude toward the cash transfer policy was coded as “y = 1” when the response was “good policy”; otherwise, it was coded as “y = 0.” We used a multivariable logistic regression model to explore the relationship between self-rated political orientation and attitude toward the cash transfer policy. The variables used for adjusting the model included gender, age, occupation, self-reported household economic status, residential area, income change during the COVID-19 pandemic, and risk perception; the occupation variable was excluded, as it was a source of multicollinearity (Supplementary Table S2). Additional models with interaction terms were fitted to identify the role of political orientation and income change during the pandemic. Model 1 featured an interaction term between risk perception and political orientation. Model 2 included a term between political orientation and income change. Model 3 had a term between risk perception and income change (Supplementary Table S3). The threshold for significance (alpha) was 5%, but we only presented estimates and 95% confidence intervals instead of reporting *p*-value. The models' goodness-of-fit statistics were calculated using Hosmer and Lemeshow test (51). The logistic regression results were presented as figures showing the average marginal effects instead of the usual odds ratio (52). All statistical analyses were performed using R Software (Version 4.0.3; R Foundation for Statistical Computing, Vienna, Austria), and average marginal effects were calculated using R package *margins*.

## Results

### Policy Process and Trends of the COVID-19 Epidemic

**Figure 1:**
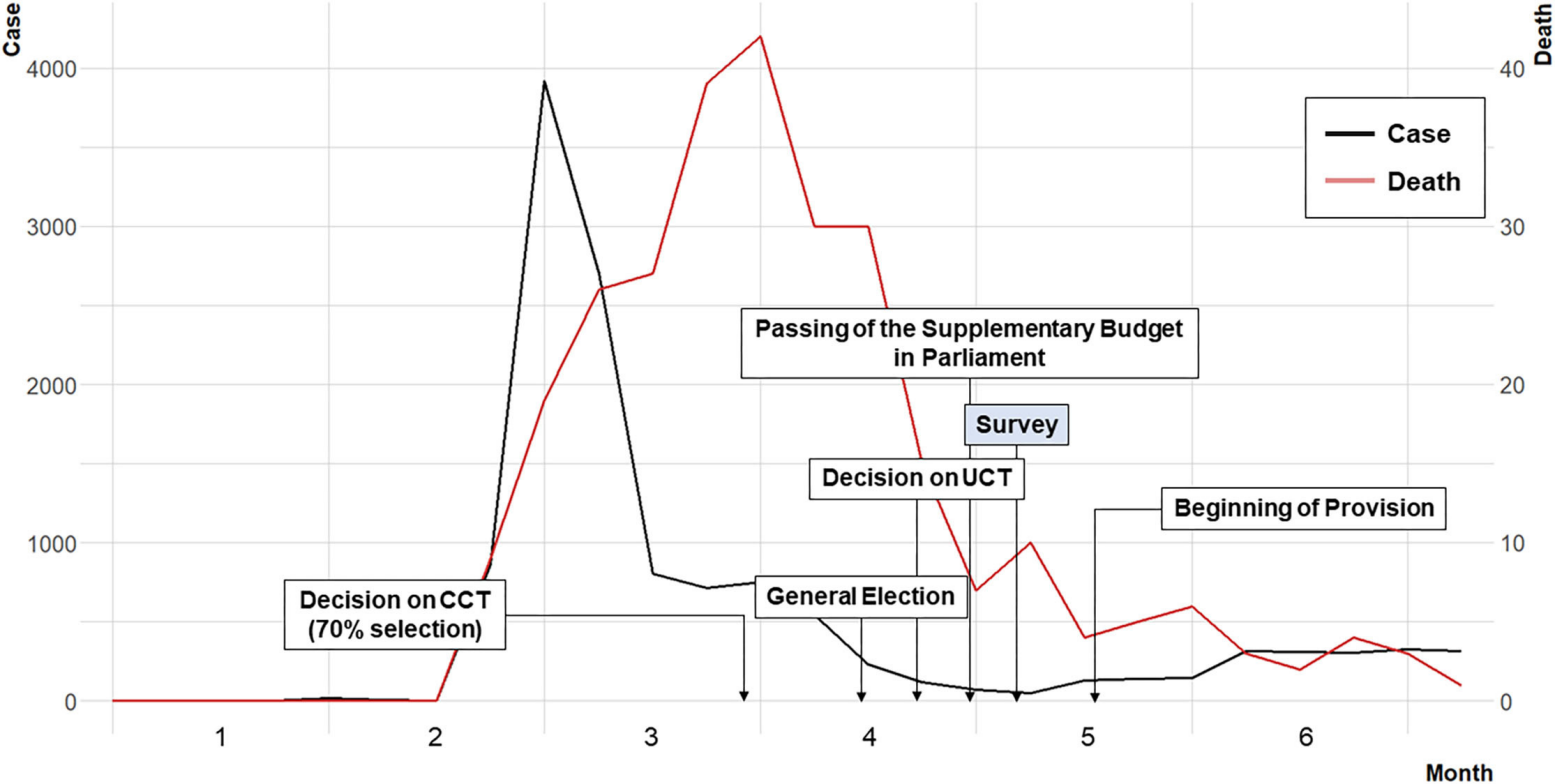
Epidemic curve and the development of the cash transfer policy discourse in 2020. The cases and deaths were calculated as seven-day moving averages, and the main events were marked in boxes. CCT, conditional cash transfer; UCT, unconditional cash transfer.

The epidemic curve and the cash transfer discourse development are shown in Figure 1. The bottom 70% payment was decided 3 weeks before the general election, and the universal payment and the accompanying supplementary budget were organized immediately after the general election.

### Demographic Factors

The participants' general characteristics are presented in Table 1. Eighty percent of the participants were favorable to the cash transfer policy, while other 20 percent responded it was a bad policy. Approximately half of the participants were female (50.4%), aged <50 years (53%), had middle-level self-reported household income (46.6%), and lived in the Seoul metropolitan area (50.1%). Over half of the participants were worried about COVID-19 infection (56%) and had not experienced income decrease during the pandemic (51.1%). Overall, 20, 45, and 35% of the sample were conservatives, moderates/non-committed, and progressives, respectively, and four-fifths felt that the cash transfer policy was reasonable. Subgroup analysis was performed to check if there is any difference in demographic factors depending on the attitude toward the policy. The results showed substantial differences between the group who thought that the cash transfer policy was a good policy and the group who felt it was a bad policy regarding age, residential area, and political orientation. Still, there was no difference in this regard among gender, self-reported household income, risk perception, and income change during the COVID-19 pandemic.

**Table 1:** Participants' basic characteristics.

**Variable**		**Attitude toward the cash transfer policy (Proportion)^a^**			
		**Total**	**Bad policy**	**Good policy**	***P*-value^b^**
Gender	Male	49.6	45.1	51.6	0.117
	Female	50.4	54.9	48.3	
Age (years)	18–29	18.1	11.7	20.1	
	30–39	15.9	12.5	17.4	
	40–49	19.0	12.1	22.0	<0.001^***^
	50–59	19.7	30.4	17.1	
	60 and older	27.2	33.2	23.3	
Self-reported household income	Upper	14.4	13.9	14.9	
	Middle	46.6	46.8	47.7	0.877
	Lower	39.0	39.3	37.3	
Residential area	Seoul metropolitan area	50.1	49.3	50.6	<0.001^***^
	Chung-chung	10.5	11.8	10.3	
	Ho-nam	9.9	3.3	12.0	
	Yeong-nam	25.2	33.6	22.3	
	Gangwon/Jeju	4.3	2.1	4.8	
Risk perception (affective)	Not worried	44.0	38.9	46.8	0.058^*^
	Worried	56.0	61.1	53.2	
Risk perception (cognitive)	Not worried	52.5	50.8	52.9	0.627
	Worried	47.5	49.2	47.1	
Income change during the COVID-19 pandemic	Decreased	48.9	49.7	49.0	0.879
	No change or increased	51.1	50.3	51.0	
Political orientation	Conservative	21.2	36.8	16.1	<0.001^***^
	Don't know/refuse to respond	16.1	16.9	15.7	
	Moderate	29.5	32.8	28.2	
	Progressive	33.2	13.4	40.1	

a *For some items, summing the constituent percentages may not produce a result of 100 because of rounding*.

b *Chi-square tests were performed to evaluate the distribution of each variable depending on attitude to the cash transfer policy*.

***, **, and * *denote statistical significance at the 1, 5 and 10% level, respectively*.

### The Relationship Between Political Orientation and Attitude Toward the Cash Transfer Policy

**Figure 2:**
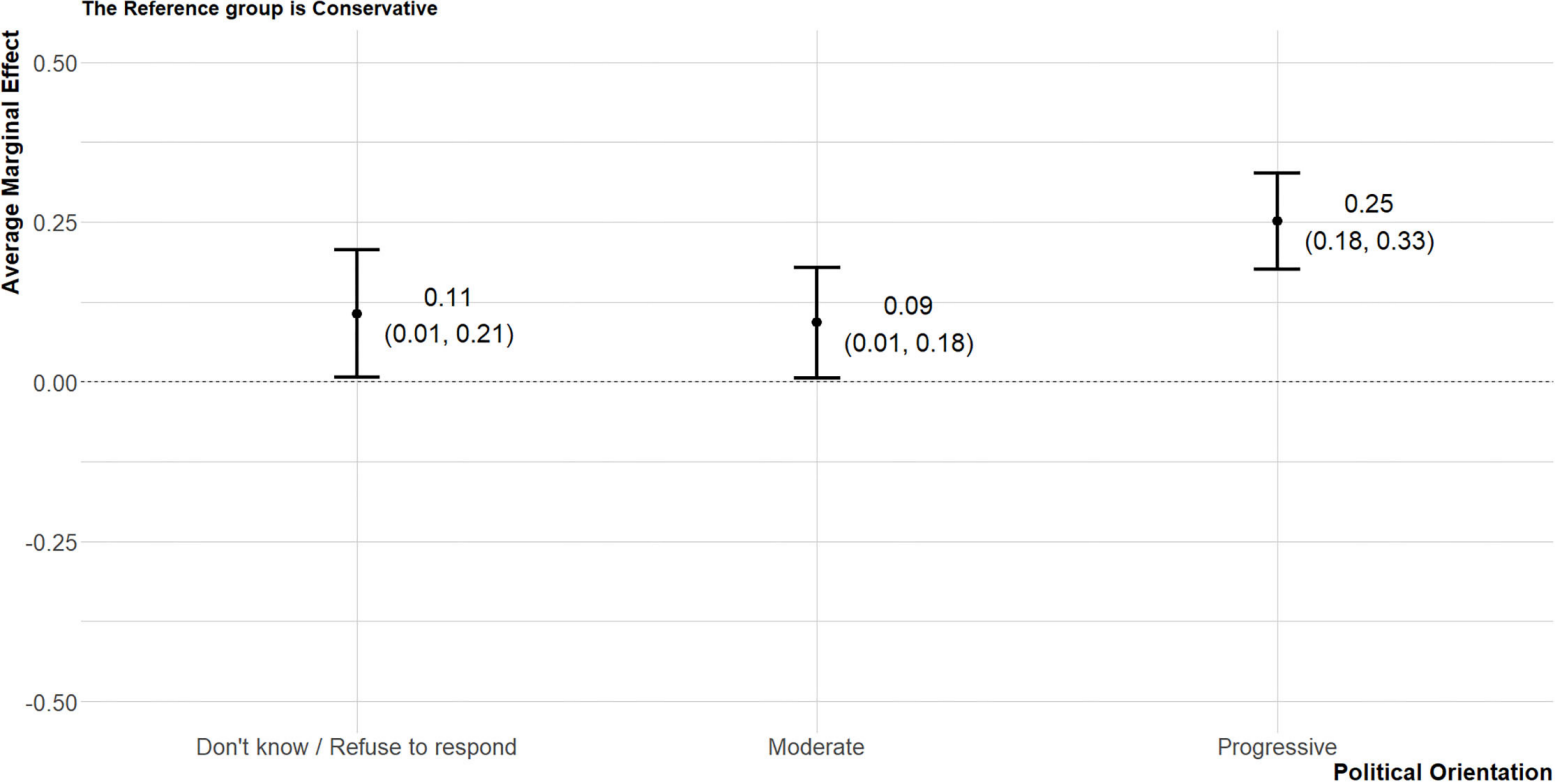
The average marginal effects of political orientation on attitude toward the cash transfer policy. The reference group is conservative.

**Figure 3:**
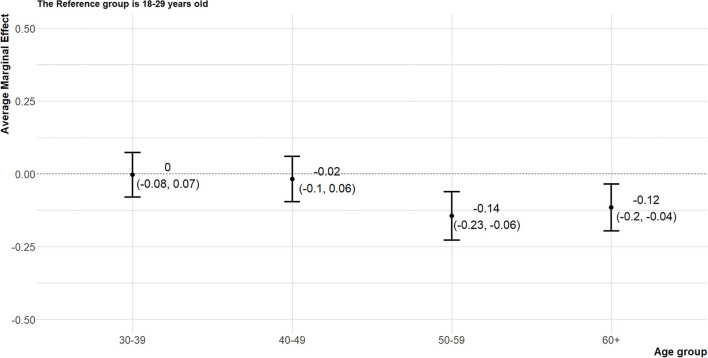
The average marginal effects of age on attitude toward the cash transfer policy. The reference group is 18–29 years old.

**Figure 4:**
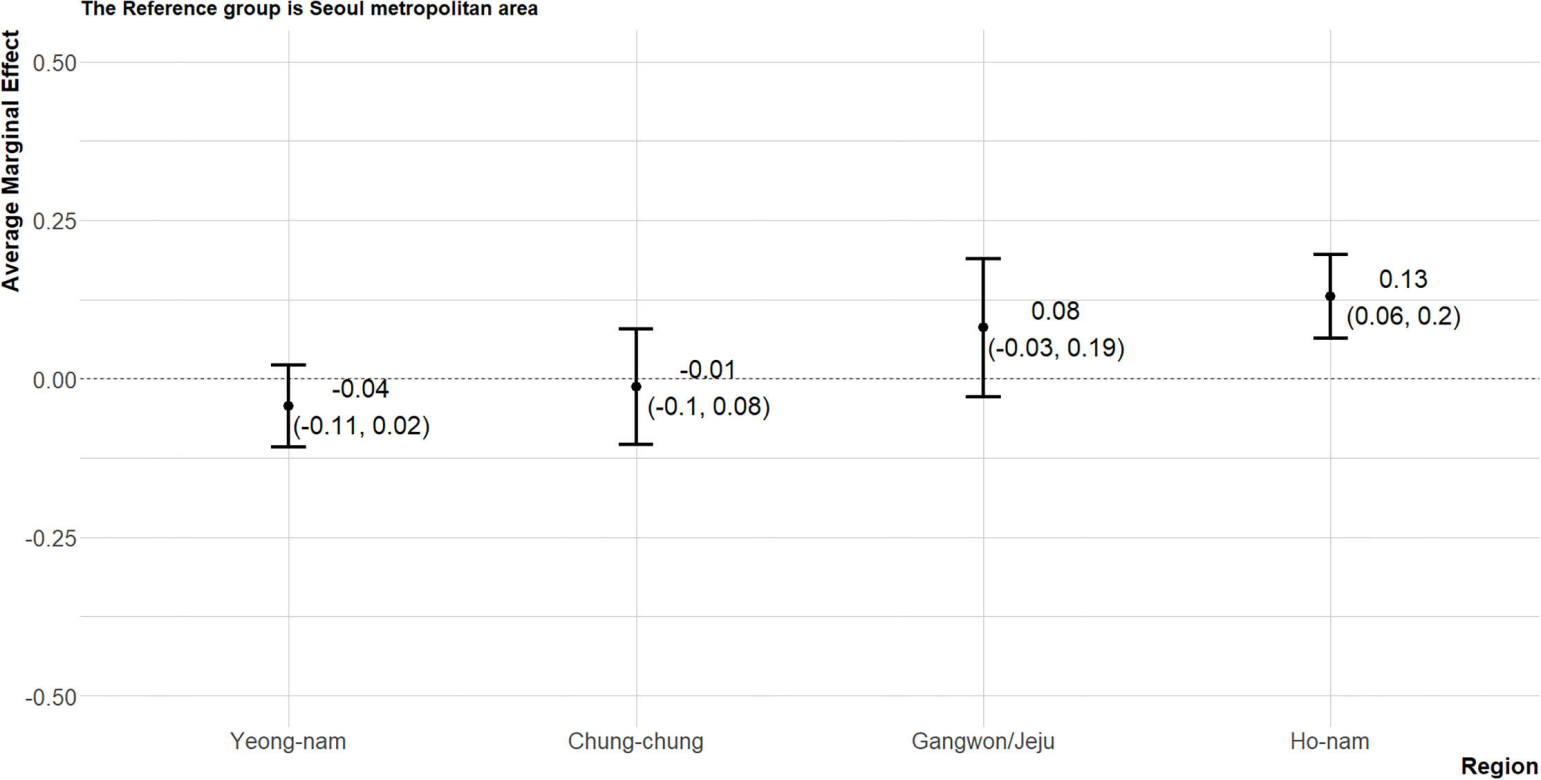
The average marginal effects of residential area on attitude toward the cash transfer policy. The reference group is Seoul metropolitan area.

The average marginal effects (AME) of political variables (political orientation, age, and residential area) on attitude toward the cash transfer policy were presented graphically. The marginal effect refers to a change in outcome (a change in the probability of supporting the cash transfer policy in the article) when the state of the variable of interest (political orientation) changes while other variables are constant. In other words, a kind of experimental situation was assumed and the effect of each variable was calculated in virtual situations. And AME means the average of these marginal effects estimated from each person (52). The Don't know/Refuse to respond group, moderate group, and progressive group were 10.6, 9.2, 25.2%p more likely to show favorable attitudes toward the policy, respectively, than the conservative group (Figure 2). The attitudes of people in their 30s and 40s did not differ significantly from the attitudes of the 18–29-year-olds; however, people in their 50s and those aged 60 and older had 14.5 and 11.6%p lower probabilities of supporting the policy, respectively (Figure 3). Compared to the Seoul metropolitan area, only residents of the Ho-nam area showed favorable attitudes toward the cash transfer policy (13.0%p). In contrast, residents of the Yeong-nam area showed relatively unfavorable attitudes toward the policy (Figure 4).

The AME of variables on attitude toward the cash transfer policy is shown in Table 2, and the adjusted odds ratios are presented in Supplementary Table S2. Lastly, the additional models with interaction terms (political orientation and risk perception, political orientation and income changes, and risk perception and income changes) produced similar results (Supplementary Table S3). The *p*-values of the Hosmer-Lemeshow test were 0.426 (main model), 0.787 (model 1), 0.441 (model 2), and 0.604 (model 3).

**Table 2:** The average marginal effect of variables on attitude toward the cash transfer policy.

**Variable**	**AME (SE)**	**95% CI**
Gender (Ref: Male)		
Female	−0.03 (0.03)	−0.09, 0.02
Age (years) (Ref: 18–29)		
30–39	−0.00 (0.04)	−0.08, 0.07
40–49	−0.02 (0.04)	−0.10, 0.06
50–59	−0.15 (0.04)^*^	−0.23, −0.06
60 and older	−0.12 (0.04)^*^	−0.20, −0.04
Self-reported household income (Ref: Upper)		
Middle	0.03 (0.04)	−0.06, 0.11
Lower	0.04 (0.04)	−0.05, 0.12
Residential area (Ref: Seoul Metropolitan area)		
Chung-chung	−0.01 (0.05)	−0.10, 0.08
Ho-nam	0.13 (0.03)^*^	0.06, 0.20
Yeong-nam	−0.04 (0.03)	−0.11, 0.02
Gangwon/Jeju	0.08 (0.06)	−0.03, 0.19
Risk perception (affective) (Ref: Not worried)		
Worried	−0.03 (0.03)	−0.08, 0.02
Risk perception (cognitive) (Ref: Not worried)		
Worried	0.00 (0.03)	−0.05, 0.05
Income change during the COVID-19 pandemic (Ref: Decreased)		
No change or increased	−0.03 (0.03)	−0.08, 0.02
Political orientation (Ref: Conservative)		
Don't know/Refuse to respond	0.11 (0.05)^*^	0.01, 0.21
Moderate	0.09 (0.04)^*^	0.01, 0.18
Progressive	0.25 (0.04)^*^	0.18, 0.33

* *denotes statistical significance at the 5% level. AME, Average marginal effect; SE, standard error; CI, confidence interval*.

## Discussion

This study aimed to explore the relationship between political orientation and attitude toward the cash transfer policy and identify the effects of other potentially associating factors. We consequently investigated the distributions of various items and analyzed the association between self-rated political orientation and attitude toward the cash transfer policy. Age, residential area, and political orientation were correlated with attitude toward the cash transfer policy, while the other examined variables were not. These results are well in line with our prediction that factors other than political ideology would not significantly impact determining stances on the policy, as the COVID-19 pandemic and consequent income loss were not enough to overcome political orientation.

Self-rated political orientation showed a relatively strong correlation with attitude toward the cash transfer policy. The moderate and non-committed groups (i.e., those who answered “don't know/refuse to respond”) held similarly favorable views toward the policy, while the progressive group showed robust support. This result seems natural because the left-leaning centrist ruling party has adopted universal basic service policies like free school meals and free medical care in its manifesto since the 2010s. The left-wing party in parliament has supported it. The support, however, is likely to be a mixture of countenance to the ruling party from relatively conservative ruling party supporters and one to the policy from more progressive people. These results correspond well to the differences in age and residential variables. Shortly before our survey, the incumbent ruling party won a landslide victory in the general election, receiving approximately three-fifths of the available seats. Most of the votes for the winning party came from the Seoul metropolitan area and the Ho-nam area. The party fared relatively poorly in the Yeong-nam area. This can explain the somewhat unfavorable attitude toward the government's cash transfer policy we observed among residents of Yeong-nam. However, the level of opposition among residents of Yeong-nam was not very strong; this may be associated with the high presidential approval rating of 71% observed in this study and the nature of the cash transfer policy itself, and even though opponents derisively referred to the fund as “helicopter money” and “buying votes (53)”. Similarly, our research's substantial differences across age groups seem natural because the opposition party has high support among people in their 50s and older. Our results are comparable to Kim et al. (54), who surveyed the cash transfer policy in the Seoul metropolitan area (Seoul, Incheon, and Gyeonggi). The study, which investigated satisfaction with an overall appraisal of the cash transfer policy, named disaster basic income, also found that the more progressive the respondent, the higher their satisfaction with and appraisal of the funding. However, unlike our study, Kim et al. found that women and people in their 30s, 40s, and 50s had a significantly favorable attitude toward the cash transfer policy. This may be because those residing in the Seoul metropolitan area have a more progressive political orientation on average when compared to the general population of South Korea (54).

Individual characteristics (other than politics-related variables) were not associated with attitudes toward the cash transfer policy. It seems reasonable to find no association between income changes during the COVID-19 pandemic and support for the policy. People generally considered COVID-19 as being similar to wartime mobilization when the survey was performed. At that time, the intensity of the pandemic had eased slightly, and the impact the non-pharmaceutical interventions were making on the economy had not appeared yet. In other words, the first cash transfer was developed and implemented when the COVID-19 pandemic had not caused notable strife; the negative impacts due to income loss were considered bearable at that time. Likewise, the risk-perception items were also not correlated with attitude toward the policy.

This was the first study in South Korea to evaluate the relationship between political orientation and attitude toward the cash transfer policy to the best of our knowledge. We found that political orientation, age, and residential area correlated with attitudes toward the policy. Using primary survey data, this study reveals the social and political dimensions of the pandemic. Specifically, it shows that political ideology can override material hardships and psychologically perceived risks if those hardships and risks are not significant enough. This study contributes to the existing literature in that it reveals that political ideology has the power to remain influential in the turmoil of the pandemic, and a sound social science approach that properly considers the political dimension of policy to support the COVID-19 pandemic response is needed (21). The political process, which includes the process of problem definition, explains most of the policy process, and policy options are to be chosen among the existing ones. The argument that a well-established good policy should be selected is normatively relevant, but it does not reflect the actual policy process well. Instead, it is a more appropriate prescription that a good policy should be embedded in the proper understanding of the political process. There may be various disagreements on whether the cash transfer policy in South Korea a good policy was. Still, as the results of this study show, it is difficult to refute the policy formation process as a political process. In other words, our research only reaffirmed the long-standing theme of health policy that policy and politics cannot be separated (55, 56).

However, there are many limitations to this research. First, we used cross-sectional survey data, and we did not have enough variables to identify and estimate causal effects. Thus, even though they may have some causal components based on theories, the observed associations can only be considered tentative in nature. Second, the public attitudes toward the survey instruments used in the study, political orientation, risk perception, and attitude to policy, are highly volatile. The survey was conducted during the “honeymoon period” that occurred shortly after the Daegu outbreak (February–March 2020) had subsided; at that time, the daily number of COVID-19 cases remained low, epidemic response policy packages (so-called “K-quarantine”) had been gaining worldwide attention, and the ruling party had won a historic victory in the general election. This context provides valuable information in interpreting our findings and makes it difficult to generalize the cash transfer policies during the disaster. Third, the survey's sample size was not sufficiently large to include the occupation variable, which we excluded due to a multicollinearity problem and may also have been too small to identify other crucial associations.

In the specific context of South Korea, this study evaluated the relationship between self-rated political orientation and attitude toward the cash transfer policy for alleviating the economic effects of the COVID-19 pandemic, and the impact of other potentially associating factors. We found that self-rated political orientation and related individual characteristics with political implications (age and residential area) were strongly associated with attitude toward the policy. We reaffirmed the political nature of the policy process and found that pursuing good policy is not much different from pursuing good politics. Although the results of this study illuminate the political and social dimensions of pandemics, we could not fully identify the determinants and mechanisms of attitudes toward policies outside the scope of traditional health policy. Further research that considers the contexts of other countries and attitudes in South Korea at different time points will generate valuable knowledge for understanding the political and social dimensions of pandemics and associated responses, and it can also help us prepare for future pandemics.

## Data Availability Statement

The data analyzed in this study is subject to the following licenses/restrictions: Data from this study cannot be publicly shared because we have used third-party data from Gallup Korea and are not entitled to share the data. Gallup Korea Daily Opinion survey is a telephone research program that has been operated weekly by Gallup Korea since January 2012. It examines basic state-run indicators, including presidential job performance evaluation and political party support, and Koreans' thoughts on major political issues, economy, society, life and culture. Results of basic analysis will be released every Friday at 10 a.m. on Gallup Korea's website (www.gallup.co.kr). Gallup Korea plans and pays for itself, and anyone interested can use the results of the survey for free. However, the use of raw data from the Gallup Korea is allowed only for researchers conducting a joint study with a Gallup Korea researcher. Detailed data approval procedures are carried out in accordance with Gallup Korea's internal guidelines. More information about sharing the data can be obtained by contacting press@gallup.co.kr. Requests to access these datasets should be directed to www.gallup.co.kr.

## Ethics Statement

This study was reviewed and approved by the Institutional Review Board (IRB) of Seoul Metropolitan Government–Seoul National University Boramae Medical Center (IRB No. 07-2021-13). As a result of the nature of this study, the need for informed consent was waived by the participants; this waiving of consent was approved by the IRB.

## Author Contributions

JHK and WMJ: conceptualization and writing—review and editing. DHJ: data curation and investigation. JHK and DHJ: formal analysis. DHJ, JHK, and WMJ: methodology. JHK: writing—original draft. All authors contributed to the article and approved the submitted version.

## Funding

JHK is supported by the National Research Foundation of Korea (BK21 Centers for Integrative Response to Health Disasters, Graduate School of Public Health, Seoul National University) (Grant No. 4199990514025; https://bk21four.nrf.re.kr/). The funder did not play any role in the study design, data collection and analysis, decision to publish, or preparation of the manuscript.

## Conflict of Interest

DHJ is affiliated with Gallup Korea (https://www.gallup.co.kr/) but did not receive any funding from them for this work. The remaining authors declare that the research was conducted in the absence of any commercial or financial relationships that could be construed as a potential conflict of interest.

## Publisher's Note

All claims expressed in this article are solely those of the authors and do not necessarily represent those of their affiliated organizations, or those of the publisher, the editors and the reviewers. Any product that may be evaluated in this article, or claim that may be made by its manufacturer, is not guaranteed or endorsed by the publisher.
