# Supplementary material for: Association Between Self-Rated Political Orientation and Attitude Toward the Cash Transfer Policy During the COVID-19 Pandemic: A Nationwide Cross-Sectional Survey Conducted in South Korea
Source: Front Public Health. 2022 May 17;10:887201. doi: 10.3389/fpubh.2022.887201 (PMC9152266; doi:10.3389/fpubh.2022.887201)
Supplement: Supplementary file 1 [file Table_1.DOCX]

Supplementary Material

# S1 File. Survey details

**Basic information**

- Registration number: 7290
- Poll Name
- Election type: other political party support
- Region: nationwide
- Election name: regular survey of party support (national regular survey of party support, 1st week of May 2020)
- Client: Surveyor’s own investigation – Gallup Korea’s own investigation
- Surveyor name: Gallup Korea
- Survey area: nationwide
- Date of survey: 2020-05-06 10:00-18:00, 2020-05-07 10:00-18:00
- Number of days surveyed: 2
- Survey time: 16 hours
- Survey subject: men and women aged 18 or older nationwide

Sample Size

| **Classification** | | **Completed cases (person)** | **Allocated cases (person)** |
| --- | --- | --- | --- |
| Total | | 1004 | 1000 |
| Sex | Male | 505 | 496 |
|  | Female | 499 | 504 |
| Age group | 18-29 | 159 | 181 |
|  | 30s | 158 | 159 |
|  | 40s | 183 | 190 |
|  | 50s | 210 | 197 |
|  | 60s or older | 294 | 272 |
| Region | Seoul | 194 | 193 |
|  | Incheon / Gyeong-gi | 309 | 309 |
|  | Gangwon | 32 | 30 |
|  | Daejeon / Sejong / Chung-chung | 103 | 105 |
|  | Gwangju / Jeolla | 99 | 99 |
|  | Daegu / Gyeong-buk | 101 | 99 |
|  | Busan / Ulsan / Gyeong-nam | 154 | 153 |
|  | Jeju | 12 | 13 |

**Survey Results**

- Weight calculation and application method
  - Basic weight
    - Calculation: Weights by sex, age, and region based on population registered by the Ministry of the Interior and Safety at the end of March 2020
    - Application: cell weighting
  - Sampling error: ±3.1% in 95% confidence interval
- Survey Results
  - (Publication media) Broadcasting (internet) newspaper and news communication
  - (Name of media) Gallup Korea homepage
  - (Date and time of initial publication) 2020-05-08 10:00 AM
