# Supplementary material for: Association Between Self-Rated Political Orientation and Attitude Toward the Cash Transfer Policy During the COVID-19 Pandemic: A Nationwide Cross-Sectional Survey Conducted in South Korea
Source: Front Public Health. 2022 May 17;10:887201. doi: 10.3389/fpubh.2022.887201 (PMC9152266; doi:10.3389/fpubh.2022.887201)
Supplement: Supplementary file 2 [file Table_2.DOCX]

**S1 Table. Proportion of missing values by variables**

| **Variable^a^** | **Proportion** |
| --- | --- |
| Self-reported household income | 1.5% |
| Risk perception (affective) | 0.8% |
| Risk perception (cognitive) | 5.9% |
| Income change during the COVID-19 Pandemic | 1.2% |

^a^ The following variables were not included because of there were no missing values: Age, Gender, Region, Political orientation, Attitude to the disaster-relief fund
