# Supplementary material for: Association Between Self-Rated Political Orientation and Attitude Toward the Cash Transfer Policy During the COVID-19 Pandemic: A Nationwide Cross-Sectional Survey Conducted in South Korea
Source: Front Public Health. 2022 May 17;10:887201. doi: 10.3389/fpubh.2022.887201 (PMC9152266; doi:10.3389/fpubh.2022.887201)
Supplement: Supplementary file 3 [file Table_3.DOCX]

Supplementary Material

# S2 File. Interview guide of the Gallup Korea

## Area

Which region do you live in? Please tell me based on your address.

*We don't have your location information because we've randomly generated your telephone number.

1. Seoul

2. Busan

3. Daegu

4. Incheon

5. Gwangju

6. Daejeon

7. Ulsan

8. Sejong

9. Gyeonggi

10. Gangwon

11. Chungbuk

12. Chungnam

13. Jeonbuk

14. Jeonnam

15. Gyeongbuk

16. Gyeongnam

17. Jeju

**Age**

Please answer your age.

**Gender**

Please answer your gender.

1. Male
2. Female

**Affective risk perception**

The new coronavirus infection, COVID-19, is spreading recently.

“How much are you worried about being infected with COVID-19?

1. Very much worried
2. Somewhat worried
3. Not so worried
4. Not worried at all

9. Don’t know/Refused

**Cognitive risk perception**

“How likely do you think you are to be infected with COVID-19?”

1. Very likely
2. Somewhat likely
3. Less likely
4. Not likely at all

9. Don’t know/Refused

**Attitude toward disaster-relief fund**

The government has decided to provide disaster-relief funding ranging from 400,000 Korean won for single-person households to one million Korean won for households with four or more people. Do you think that this is a good policy or a bad policy?

1. Good policy
2. Bad policy

9. Don’t know/Refused

**Income change during the COVID-19 Pandemic**

How has your household income changed in the past month compared to the previous one due to COVID-19?

1. Decreased
2. Increased
3. No change

9. Don’t know/Refused

**Occupation**

What is your occupation?

1. Farming/forestry/fishery
2. Self-employed
3. Blue collar (sales/services, functional/skilled workers, general workers)
4. White collar (office/technical position, management, professional/freelancer)
5. Homemaker
6. Student
7. Unemployed
8. Retired
9. Other (Specify: _____________ )

99. Don’t know/Refused

**Political self-identification**

Which of the following best describes your political tendency?

1. Very conservative
2. Conservative
3. Moderate
4. Liberal
5. Very liberal

9. Don’t know/Refused

**Self-reported household economic status**

“If you divide the standard of living of Korean people into five levels: Upper, Upper middle, Middle, Lower middle, and Lower, which level do you think your household`s living standard belongs to?”

1. Upper
2. Upper middle
3. Middle
4. Lower middle
5. Lower

9. Don’t know/Refused

The results of the survey were published on the Gallup Korea website (www.gallup.co.kr). In Korea, opinion polls, which include party support, are classified as election surveys, and were registered on the website of the National Election Survey Deliberation Commission (www.nesdc.go.kr) according to the regulations.
