# Supplementary material for: Association Between Self-Rated Political Orientation and Attitude Toward the Cash Transfer Policy During the COVID-19 Pandemic: A Nationwide Cross-Sectional Survey Conducted in South Korea
Source: Front Public Health. 2022 May 17;10:887201. doi: 10.3389/fpubh.2022.887201 (PMC9152266; doi:10.3389/fpubh.2022.887201)
Supplement: Supplementary file 4 [file Table_4.DOCX]

**S2 Table. Analysis of factors potentially associated with attitude towards the cash transfer policy.**

| **Variables** | | **aOR (95% CI)** |
| --- | --- | --- |
| Gender | Male | 1.00 |
|  | Female | 0.78 (0.54–1.13) |
| Age (years) | 18–29 | 1.00 |
|  | 30–39 | 0.97 (0.47–2.00) |
|  | 40–49 | 0.85 (0.41–1.72) |
|  | 50–59 | 0.36 (0.18–0.66) |
|  | 60 and older | 0.42 (0.22–0.79) |
| Self-reported household income | Upper | 1.00 |
|  | Middle | 1.22 (0.68–2.13) |
|  | Lower | 1.34 (0.73–2.42) |
| Residential area | Seoul metropolitan area | 1.00 |
|  | Chung-chung | 0.92 (0.50–1.75) |
|  | Ho-nam | 3.76 (1.58–11.17) |
|  | Yeong-nam | 0.75 (0.50–1.15) |
|  | Gangwon/Jeju | 1.97 (0.72–6.94) |
| Risk perception (affective) | Not worried | 1.00 |
|  | Worried | 0.80 (0.54–1.18) |
| Risk perception (cognitive) | Not worried | 1.00 |
|  | Worried | 0.80 (0.68–1.48) |
| Income changes during the COVID-19 Pandemic | Decreased | 1.00 |
|  | No change or increased | 0.81 (0.55–1.19) |
| Political orientation | Conservative | 1.00 |
|  | Don’t know/Refuse to respond | 1.79 (1.02–3.19) |
|  | Moderate | 1.65 (1.04–2.61) |
|  | Progressive | 6.80 (3.89–12.33) |
| **aOR: adjusted OR; CI: confidence interval** | | |
