# Supplementary material for: Association Between Self-Rated Political Orientation and Attitude Toward the Cash Transfer Policy During the COVID-19 Pandemic: A Nationwide Cross-Sectional Survey Conducted in South Korea
Source: Front Public Health. 2022 May 17;10:887201. doi: 10.3389/fpubh.2022.887201 (PMC9152266; doi:10.3389/fpubh.2022.887201)
Supplement: Supplementary file 5 [file Table_5.DOCX]

Supplementary Material

# S3 File. Approach for surveying affective and cognitive risk perception

The survey examined two aspects of risk perception: affective and cognitive. Affective risk perception was assessed using the question: “How worried are you that you could contract COVID-19?” Responses were recorded using a 4-point scale, where “4” represented “very much worried” and “1” represented “not worried at all”; the responses were then reclassified as follows: 1–2 = “not worried,” 3–4 = “worried.” The proportion of affective risk perception was defined as the ratio of the number of participants who were “worried” to the number of eligible respondents. Cognitive risk perception was evaluated using the question: “Do you think there is a possibility that you will contract COVID-19?” Responses for cognitive risk perception were assessed and reclassified in the same way as those for affective risk perception.
