# Supplementary material for: Association Between Self-Rated Political Orientation and Attitude Toward the Cash Transfer Policy During the COVID-19 Pandemic: A Nationwide Cross-Sectional Survey Conducted in South Korea
Source: Front Public Health. 2022 May 17;10:887201. doi: 10.3389/fpubh.2022.887201 (PMC9152266; doi:10.3389/fpubh.2022.887201)
Supplement: Supplementary file 6 [file Table_6.DOCX]

**S3 Table. Analysis of factors potentially associated with attitude towards the cash transfer policy. (Additional models).**

| **Variables** | | **Model 1** | **Model 2** | | | **Model 3** |
| --- | --- | --- | --- | --- | --- | --- |
|  |  | **aOR**  **(95% CI)** | **aOR**  **(95% CI)** | | | **aOR**  **(95% CI)** |
| Gender | Male | 1.00 | 1.00 | | | 1.00 |
|  | Female | 0.78  (0.54–1.14) | 0.78  (0.53–1.13) | | | 0.78  (0.54–1.14) |
| Age (years) | 18–29 | 1.00 | 1.00 | | | 1.00 |
|  | 30–39 | 0.94  (0.45–1.95) | 0.96  (0.46–1.99) | | | 0.98  (0.47–2.02) |
|  | 40–49 | 0.82  (0.40–1.66) | 0.86  (0.42–1.75) | | | 0.87  (0.42–1.78) |
|  | 50–59 | 0.34  (0.17–0.64) | 0.35  (0.18–0.66) | | | 0.37  (0.19–0.68) |
|  | 60 and older | 0.41  (0.21–0.77) | 0.42  (0.22–0.78) | | | 0.40  (0.22–0.81) |
| Self-reported  household income | Upper | 1.00 | 1.00 | | | 1.00 |
|  | Middle | 1.23  (0.68–2.16) | 1.21  (0.67–2.12) | | | 1.24  (0.69–2.17) |
|  | Lower | 1.38  (0.75–2.50) | 1.34  (0.73–2.42) | | | 1.36  (0.74–2.45) |
| Residential area | Seoul metropolitan area | 1.00 | 1.00 | | | 1.00 |
|  | Chung-chung | 0.89  (0.49–1.71) | 0.91  (0.49–1.73) | | | 0.89  (0.48–1.70) |
|  | Ho-nam | 3.83  (1.60–11.41) | 3.73  (1.56–11.09) | | | 3.74  (1.56–11.14) |
|  | Yeong-nam | 0.76  (0.50–1.17) | 0.75  (0.49–1.15) | | | 0.76  (0.50–1.16) |
|  | Gangwon/Jeju | 2.08  (0.76–7.31) | 2.07  (0.75–7.32) | | | 1.92  (0.71–6.77) |
| Risk perception  (affective) | Not worried | 1.00 | 1.00 | | | 1.00 |
|  | Worried | 1.08  (0.55–2.13) | 0.80  (0.54–1.18) | | | 0.80  (0.46–1.40) |
|  | Interaction with political orientation | | | | | |
|  | - Don’t know/Refuse to respond | 1.34  (0.39–4.54) | | - | - | |
|  | - Moderate | 0.64  (0.25–1.60) | | - | - | |
|  | - Progressive | 0.30  (0.08–1.09) | | - | - | |
|  | Interaction with income changes during the COVID-19 Pandemic | | | | | |
|  | - No change or increased | - | | - | 1.00  (0.46–2.19) | |
| Risk perception  (cognitive) | Not worried | 1.00 | | 1.00 | 1.00 | |
|  | Worried | 0.97  (0.50–1.89) | | 1.02  (0.70–1.51) | 0.77  (0.45–1.33) | |
|  | Interaction with political orientation | | | | | |
|  | - Don’t know/Refuse to respond | 0.80  (0.24–2.70) | | - | - | |
|  | - Moderate | 1.29  (0.52–3.23) | | - | - | |
|  | - Progressive | 0.90  (0.26–3.02) | | - | - | |
|  | Interaction with income change during the COVID-19 Pandemic | | | | | |
|  | - No change or increased | - | | - | 1.71  (0.79–3.73) | |
| Income change  during  the COVID-19 Pandemic | Decreased | 1.00 | | 1.00 | 1.00 | |
|  | No change or increased | 0.80  (0.54–1.17) | | 0.82  (0.42–1.59) | 0.63  (0.33–1.17) | |
|  | Interaction with political orientation | | | | | |
|  | - Don’t know/Refuse to respond | - | | 1.51  (0.49–4.75) | - | |
|  | - Moderate | - | | 0.83  (0.33–2.06) | - | |
|  | - Progressive | - | | 0.92  (0.28–2.86) | - | |
| Political orientation | Conservative | 1.00 | | 1.00 | 1.00 | |
|  | Don’t know/Refuse to respond | 1.61  (0.65–4.16) | | 1.45  (0.69–3.18) | 1.88  (1.07–3.37) | |
|  | Moderate | 1.90  (0.89–4.06) | | 1.81  (0.97–3.44) | 1.67  (1.06–2.66) | |
|  | Progressive | 15.36  (5.45–51.63) | | 7.12  (3.21–17.57) | 6.92  (3.95–12.56) | |

aOR: adjusted OR; CI: confidence interval
